# Nanostructured polyurethane perylene bisimide ester assemblies with tuneable morphology and enhanced stability

**DOI:** 10.1098/rsos.171686

**Published:** 2018-03-21

**Authors:** Xiaoxiao Zhang, Tingyuan Gong, Hong Chi, Tianduo Li

**Affiliations:** Shandong Provincial Key Laboratory of Fine Chemicals, School of Chemistry and Pharmaceutical Engineering, Qilu University of Technology, Jinan 250353, People's Republic of China

**Keywords:** polyurethane, perylene bisimide, self-assembly, fluorescence, thermal stability

## Abstract

Size control has been successfully achieved in inorganic materials, but it remains a challenge in polymer nanomaterials due to their polydispersity. Here, we report a facile approach to tailor the diameters of polyurethane (PU) nanoparticles (490 nm, 820 nm and 2.1 µm) via perylene bisimide (PBI) assisted self-assembly. The formed morphologies such as spindle, spherical and core–shell structures depend on the ratio of PBI and polymer concentrations. It is shown that the formation of PU nanoparticles is directed by π–π stacking of PBI and the morphology transition is not only affected by the amount of PBI incorporated, but also influenced by solvent, which controls the initial evaporation balance. Furthermore, the prepared PUs exhibit retained optical stability and enhanced thermal stability. The PUs, designed to have conjugated PBI segments in backbones, were synthesized via ring-opening and condensation reactions. Compared with the neat PU, gel permeation chromatography shows narrower molecular weight distribution. Fluorescence spectra and ultraviolet–visible spectra indicate retained maximum emission wavelength of PBI at 574 nm and 5.2% quantum yields. Thermo-gravimetric analysis and differential scanning calorimetry reveal 79°C higher decomposition temperature and 22°C higher glass transition temperature. This study provides a new way to fabricate well-defined nanostructures of functionalized PUs.

## 1. Introduction

Polyurethanes (PUs) have been widely used in medical, automotive, packaging, automobile, coating and consumer care products because of their unique characteristics such as biodegradability and mechanical properties [1,2]. To endow PUs with specific performances, a growing number of efforts either on structures or morphologies have been contributed. Mya *et al*. [3] and Wei *et al*. [4] reported polyhedral oligomeric silsesquioxane-grafted PUs which showed enhanced thermal stability and mechanical properties because of the rigid intrinsic property of silsesquioxanes. Rao *et al*. [5] reported triazine functionalized PU exhibiting luminous properties and improved mechanical properties. Fang *et al*. [6] prepared fluorine-modified PU, and the obtained PU films showed self-healing ability due to the presence of F element. Meanwhile, attempts have also been made by introducing special segments in PUs. The resulting amphiphilic structures of PUs have gained increasing attention in the fields of nanomedicine, microreactor and biomimicry [7–9]. Several types of nanostructures such as spherical, colloidal particles, worm-like micelles and vesicles have been successfully prepared in the last decade. Among these, Savić *et al*. [10] reported that amphiphilic block PUs prepared by poly(caprolactone)-*b*-poly(ethylene oxide) and tetramethylrhodamine-5-carbonyl azide could self-assemble into spherical nanoparticles (10–100 nm) in aqueous media. Zhang *et al*. [11] synthesized amphiphilic multiblock poly(lactic acid)–PU containing carboxylic groups in the main chain with 2,2-dimethylolpropionic acid as raw materials. The obtained PUs could form stable micelles through the interaction between hydrophilic poly(ethylene glycol) (PEG) segments, carboxylic groups and hydrophobic poly(lactic acid) chains in water. Cheng *et al*. [12] reported phosphate ester incorporated PUs (PUP). These amphiphilic PUPs could self-assemble into spherical, worm-like micelles, vesicles and large vesicles in aqueous solution controlled by the hydrophilic phosphates that interrupted the electrostatic force balance between chains. Although many efforts have been made in modifying the polymer structure to obtain desired nano-morphologies, problems still exist in size control and dispersion which extremely limit high performance applications.

Perylene bisimide (PBI) and its derivatives are a kind of organic dye with excellent optical properties, thermal stability and high molar absorptivity, which draw much attention in the fields of *n*-type channel of organic field-effect transistors [13], light-harvesting systems [14], bio-/chemosensors [15,16], organic solar cells [17], organic light-emitting diodes and organic lasers [18]. The planar structure with delocalized π bonds could promote intermolecular interactions with neighbouring molecules through π–π stacking [19]. In line with these properties, various PBI assemblies and their photophysics were investigated [19,20]. Self-assemblies of PBI derivatives have attracted much attention in terms of organic electronics, photonics and sensing [21]. However, either fluorescence emission of the PBI derivatives changes or the quantum yield greatly decreases in most systems due to self-quenching problem after assembly.

Here, ε-caprolactone was used as a chain extender to graft onto PBI via ring-opening reactions. The branched chains of polymer are expected to separate emission groups both intermolecularly and intramolecularly [22] and retain fluorescence properties of PBI after assembly. A series of PU perylene bisimide esters (PBI-PUs) were obtained through the ring-opening reaction and the condensation method. The structure of polymers was characterized by ^1^H nuclear magnetic resonance (^1^H NMR), Fourier transform infrared spectroscopy (FTIR) and gel permeation chromatography (GPC). The self-assembled morphologies of PBI-PUs were observed by scanning electron microscopy (SEM) and transmission electron microscopy (TEM). In addition, the thermal properties were also studied by thermogravimetric analysis (TGA) and differential scanning calorimetry (DSC). Assembled behaviour of PUs was investigated via X-ray diffraction (XRD) and the optical properties were characterized using UV–visible (UV–vis) spectroscopy and fluorescence spectroscopy.

## 2. Material and methods

### 2.1. Materials

3,4,9,10-Perylenetetracarboxylic dianhydride (98%), ε-caprolactone (99%) (ε-CL), tin(II) 2-ethylhexanoate (Sn(Oct)_2_) were all obtained from Alfa Aesar. Hexamethylene diisocyanate (HDI), diglycolamine and PEG (*M*_w_ = 1500) were purchased from Aladdin. All solvents were purchased from Sino-pharm Chemical Reagent Co. Ltd. Toluene was distilled before use, and other chemicals were used without further purification.

### 2.2. Synthesis of *N*,*N*′-bis(2-[2-hydroxyethoxy]ethyl)perylene-3,4,9,10-tetracarboxylic acid bisimide

3,4,9,10-Perylenetetracarboxylic dianhydride (2.04 g, 5.0 mmol) and diglycolamine (5.04 g, 47 mmol) were placed in a three-necked flask equipped with a thermometer and a mechanical stirrer under nitrogen atmosphere at 130°C for 3 h. After the reaction, the mixture was cooled to room temperature and diluted with ethanol (20 ml). The precipitate was obtained through centrifugation and purified with methanol (4 × 20 ml). Finally, the products were dried for 24 h at 60°C in vacuum and 6.1 g of product was obtained with yield of 86%. FTIR (KBr, cm^−1^): *ν*  =  1050 (–C–O–C–), 1653 (–C=O), 3457 (–OH).

### 2.3. Synthesis of O-2000, O-4000 and O-10000

The PBI oligomers (O-2000, O-4000 and O-10000, where the number indicates the length of poly(ε-caprolactone) (PCL)) were synthesized from PBI-OH (*N*,*N*′-bis(2-[2-hydroxyethoxy]ethyl)perylene-3,4,9,10-tetracarboxylic acid bisimide) and ε-CL through controlling the content of ε-CL. O-4000 was chosen as an example. In a typical reaction, PBI-OH (0.566 g, 1 mmol), ε-CL (3.51 g, 30 mmol), Sn(Oct)_2_ (160 µl, as catalyst) and anhydrous toluene (60 ml) were mixed into a three-necked round bottom flask. The mixture was vigorously stirred at room temperature for 5 min and then stirred at 110°C for another 20 h under dry nitrogen. After that, the solvent was removed via rotary evaporation and the mixture was precipitated out with an excess amount of methanol. Then, the precipitate was obtained through centrifugation and further washed three times using methanol. The product was obtained after drying in vacuum oven at 40°C for 24 h and 3.5 g of product was obtained with yield of 85%. ^1^H NMR (ppm, CDCl_3_): 1.4–1.6 (m, –CH_2_CH_2_CH_2_–), 2.3 (t, –COOCH_2_–), 4.1 (t, –CH_2_OH–), 8.6–8.8 (m, aromatic protons). FTIR (KBr, cm^−1^): *ν *= 1050 (–C–O–C), 1653 (–C=O), 1730 (–COO). GPC (polystyrene standard, tetrahydrofuran (THF)): *M*_w_ = 4200, *M*_w_/*M*_n_ = 1.07. O-2000 and O-10000 were prepared in the same manner.

### 2.4. Synthesis of polyurethane and perylene bisimide ester-polyurethanes

In a typical procedure of PBI-PUs (PU-2000, PU-4000 and PU-10000 where the number indicates the corresponding oligomers used), O-4000 (10 g, 2.5 mmol) was dissolved in toluene and refluxed at 110°C for 4 h. After cooling to 70°C, Sn(Oct)_2_ (0.5 wt%, with respect to reactants) was added into solution and HDI (0.412 g, 2.5 mmol) in anhydrous toluene (10 ml) was added dropwise to the solution stirring at 70°C for another 3 h under dry nitrogen. Then, 100 ml of methanol was added into the reaction mixture and stirred for another 1 h. After the reaction, solvent was removed by rotary evaporation and PU-4000 was collected by precipitation in a large amount of ethyl ether. Finally, the product was obtained after drying at 40°C for 24 h in vacuum oven and 8.9 g of product was obtained with yield of 85%. PU-2000 and PU-10000 were prepared in the same manner with yields of 87% and 86%, respectively. Neat PU was obtained using PEG and HDI in the same way.

### 2.5. Characterization

^1^H NMR spectra were recorded on a Bruker AVANCE II 400 spectrometer using tetramethylsilane (*d* = 0 ppm) as the internal standard. Chemical shifts were reported in parts per million (ppm) and CDCl_3_ was used as a solvent for all the samples. FTIR spectra were taken at room temperature via an IR Prestige-21 spectrometer (Bruker) operating at a resolution of 4 cm^−1^ with scan number 32. Infrared measurements with KBr pellet were performed within the range 4000–400 cm^−1^ on a Bruker ALPHA FTIR. XRD patterns were obtained with a D8 ADVANCE (Bruker AXS). The diffraction intensity of Cu K*α* radiation was measured in a 2*θ* range between 5° and 30° at a sweeping rate of 0.1° s^−1^. The step size was 0.033°. TGA (SDT Q600) was performed from room temperature to 1000°C at 10°C min^−1^ under air atmosphere. The thermal behaviour of polymers was analysed by DSC with a Q10 differential scanning calorimeter (TA Instruments, USA). The polymers were heated from room temperature to 150°C with a heating rate of 15°C min^−1^ under nitrogen atmosphere. Molecular weights and molecular weight distribution of the polymers were determined by GPC using THF as the eluent and polystyrene as standard. UV–vis absorption spectra were recorded on a UV-2500 spectrophotometer. The fluorescence spectra were measured with a Hitachi F-4600 fluorescence spectrophotometer. The morphologies of self-assemblies were characterized by SEM (QUANTA 200) and TEM (JEM-2100). Optical microscope (Axio Scope.A1) was used to obtain optical microscopic images.

## 3. Results and discussion

### 3.1. Synthesis and characterization of the perylene bisimide ester-polyurethanes

**Scheme 1.**
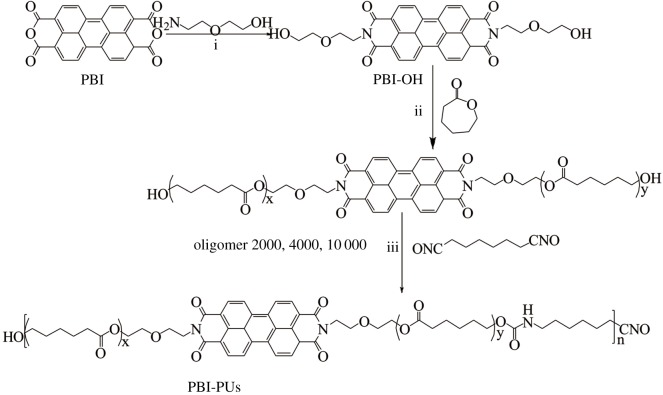
Synthetic route of PBI-PUs. (i) 130°C, 3 h; (ii) Sn(Oct)_2_, anhydrous toluene, 110°C, 20 h; and (iii) Sn(Oct)_2_, anhydrous toluene, 70°C, 3 h.

The synthetic route of PBI-PUs is shown in scheme 1. To adjust the ratio of PBI incorporated into the main chain, ε-CL was grafted onto PBI-OH first and used as a chain extender. The names of oligomers were defined according to the calculated ε-CL. To synthesize linear PUs, the oligomers were further reacted with HDI by strictly controlling ratio and reaction temperature. Finally, three kinds of PUs with different physical properties as indicated in electronic supplementary material, table S1, are obtained.

GPC shows narrow molecular weight distribution in all PBI-PUs ranging from 1.03 to 1.10 (electronic supplementary material, table S1). Here, GPC trace of PU-10000 is selected as an example shown in electronic supplementary material, figure S1. This is possibly due to the ring-opening reaction from ε-CL which possesses unique monomer length and reactive sites. Additionally, PU-4000 displays the highest molecular weight which is probably due to the high steric hindrance when a higher ratio of PBI or longer ε-CL chains are used.

The peaks in ^1^H NMR spectrum of O-2000 (electronic supplementary material, figure S2) appearing at 4.1 ppm (e), 2.3 ppm (a), 1.6 ppm (b, d), 1.4 ppm (c) are assigned to CH_2_ on PCL, suggesting that PCL is grafted onto PBI-OH successfully. The signals of PU-2000 (electronic supplementary material, figure S3) appearing at 1.4 ppm (h, i, j), 1.6 ppm (k, g) and 3.6 ppm (f) are assigned to CH_2_ protons in HDI. The typical aromatic protons of PBI appeared at 8.6–8.8 ppm in the polymers, indicating that the PBI moieties have been successfully incorporated into the main chain. The integration ratio calculation between m, n and a, b, c, d is 1.03 : 8.06 which is quite close to the feeding ratio as expected, indicating the successful polymerization between O-2000 and HDI.

FTIR of PU-2000 (electronic supplementary material, figure S4) showed that the peak of –NCO in HDI at 2260 cm^−1^ disappeared after polymerization compared with corresponding oligomer and polymer. The strong band at 1653 cm^−1^ is attributed to the CO vibrations in PBI-OH and it is recognizable in oligomer and polymer. Furthermore, the appearance of vibrational peaks of –COO at 1730 cm^−1^ and the –NH vibrations at 1536 cm^−1^ further confirms the successful polymerization.

### 3.2. Optical properties of perylene bisimide ester-polyurethanes

To optimize the concentration for self-assembly, optical spectra of PBI-PUs were obtained, both UV–vis and fluorescence spectra. The photoluminescent spectra of PBI derivatives generally show three peaks at 520–600 nm and the characteristic emission peaks normally red shift due to the serious π–π stacking and crystallization problem [23]. Here, PU-2000 is selected as a model to study the optical behaviour of PBI-PUs at different concentrations and find out the critical concentration of stacking in solution.

**Figure 1.**
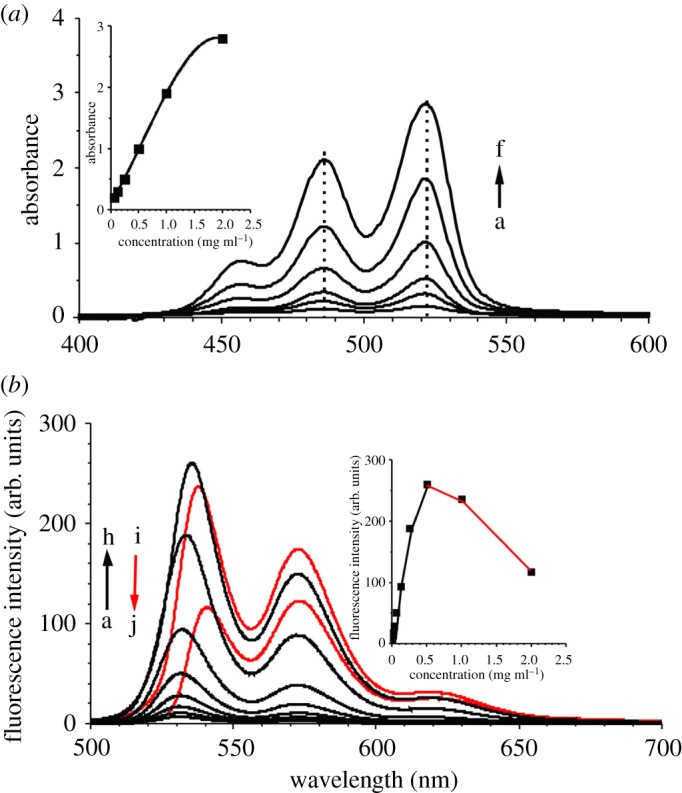
UV–vis absorption spectra of THF solution of PU-2000 at concentrations (a–f) of 0.06 mg ml^−1^, 0.125 mg ml^−1^, 0.25 mg ml^−1^, 0.5 mg ml^−1^, 1.0 mg ml^−1^ and 2.0 mg ml^−1^, respectively (*a*). Fluorescence spectra of THF solution of PU-2000 at concentrations (a–j) of 0.003, 0.06, 0.125, 0.25, 0.5, 1.0 and 2.0 mg ml^−1^ (*b*).

UV–vis spectra in figure 1*a* show the appearance of three peaks at 459, 490 and 525 nm in different concentrations ranging from 0.06 to 2.0 mg ml^−1^, which correspond to the 0–2, 0–1 and 0–0 electronic transitions of PBI [24]. What is more, the intensity of the 0–0 band is higher than that of the 0–1 band, indicating that the Franck–Condon factors favour the lower (0–0) excited vibronic state, and suggesting the absence of obvious interaction between the PBI units. A linear relation of absorption intensity to concentration is found in the range of 0.06 to 2.0 mg ml^−1^ as shown in the inset in figure 1*a*. The linear increasing tendency suggests that the solubility of PBI is greatly improved, and PU-2000 can restrain the aggregation of PBI cores through side chain effect.

Different from UV–vis results, the fluorescence intensity increases linearly with the concentration ranging from 0.003 to 0.5 mg ml^−1^ as shown in figure 1*b*. The fluorescence spectra show three bands (530–630 nm) which match well with the absorption spectra, and there was good mirror-image symmetry between the absorption and fluorescence spectra. However, the intensity persistently decreases when concentration is further increased from 0.5 to 2.0 mg ml^−1^ due to self-quenching and π–π stacking interactions occurring in the perylene cores [24,25], and the peaks are gradually shifted to the red with increasing concentration because of re-absorption [26]. The results of UV–vis and fluorescence spectra show demonstrate that these systems are in a non-aggregated state. Therefore, 0.5–2.0 mg ml^−1^ is selected for self-assembly. Interestingly, the peak only red shifts 6 nm, indicating that the optical property is retained in PU-2000. Comparable results of PU-4000 and PU-10000 are also obtained (electronic supplementary material, figures S5 and S6). We also obtained the UV–vis and fluorescence spectra of PBI-OH at concentrations of 0.003, 0.006, 0.015, 0.03, 0.06, 0.125, 0.25, 0.5 and 1.0 mg ml^−1^. As shown in electronic supplementary material, figure S7, UV–vis spectra exhibit no obvious bathochromic shift and fluorescence spectra show that the peak only red shifts 5 nm with the increase of concentration. The UV–vis and fluorescence spectra of PBI-OH are similar to those of PU-2000, PU-4000 and PU-10000, which further proves that there is no aggregation of PBI-OH and polymers in THF solution.

Photographs of PU-2000, PU-4000 and PU-10000 in THF solution under visible light and UV light with the concentration of 1.0 mg ml^−1^ are shown in electronic supplementary material, figure S8. All solutions exhibit bright greenish yellow fluorescence colour under UV excitation. This is quite different from the red colour emission in aggregation state of PBIs [27]. Relative fluorescence quantum yields of the polymers are calculated using coumarin (*Φ*_r_ = 0.56), which is dissolved in ethanol as a reference. It is calculated according to the following equation: Φ_s_ = Φ_r_·*A*_r_*n*_s_^2^*F*_s_/*A*_s_*n*_r_^2^*F*_r_, where *A*_r_ and *A*_s_ represent the absorbance at the excitation wavelength of the reference sample and the sample under test, *n*_r_ and *n*_s_ are the refractive index of the reference sample and the sample to be measured in solution, *F*_r_ and *F*_s_ are the fluorescence spectral integral area of reference and sample. Refractive index of ethanol and THF is 1.362 and 1.404, respectively. The results are 2.1%, 4.3% and 5.2% for PU-2000, PU-4000, PU-10000, respectively. PU-10000 shows the highest fluorescence quantum yield which is probably because the long and branched PCL chains could effectively separate PBI. The results further indicate that the optical properties are retained in all PBI-PUs.

### 3.3. Self-assembly of PU-2000, PU-4000 and PU-10000

**Figure 2.**
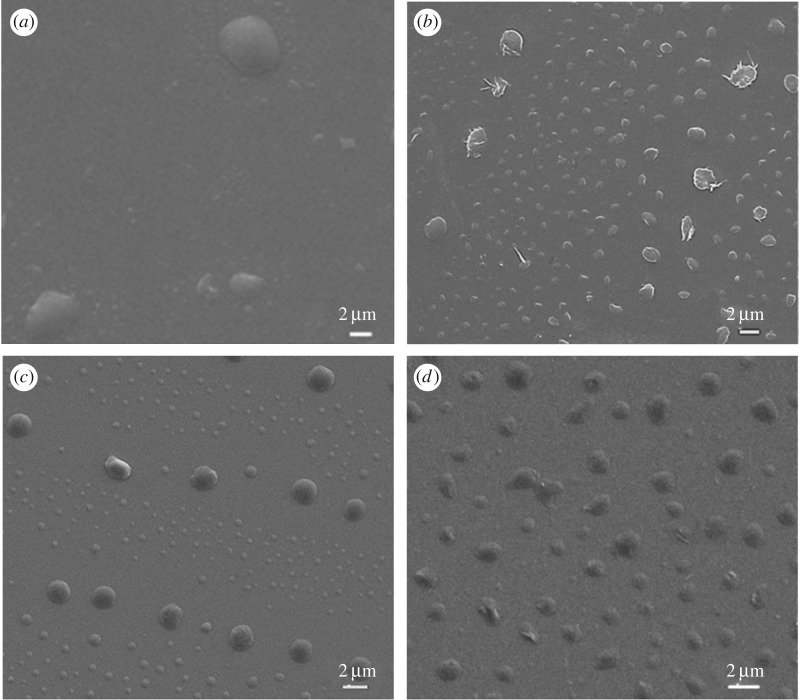
SEM images of PU (*a*), PU-2000 (*b*), PU-4000 (*c*) and PU-10000 (*d*) assembled on silicon wafer from 0.5 mg ml^−1^ mixed solutions of toluene and hexane (3 : 1, v/v).

The PBI-PUs were dispersed in mixed solvent of toluene and hexane (3 : 1, v/v) by stirring overnight at room temperature. Here, toluene was used as good solvent with higher polarity and boiling temperature. Hexane was used as poor solvent with low polarity and boiling temperature. After that, the solution was dropped onto silicon wafer and left undisturbed until dry. Compared with irregular structures in neat PU (figure 2*a*), PU-2000 forms a spindle structure with diameters of 410 nm and 1.6 µm (figure 2*b*). Interestingly, in PU-4000, the polymers spontaneously form uniform spherical structures and the diameters are approximately 490 nm and 2.1 µm in alternated arrangement (figure 2*c*). PU-10000 forms semi-spherical structure and the diameter is approximately 890 nm (figure 2*d*). The results of SEM indicate that PBI ratio plays a significant part in the assembly and morphology control of PBI-PUs. The spindle-like structure in figure 2*b* indicates rigid intrinsic property of PU-2000, while the semi-spherical structure in figure 2*d* indicates soft nature of PU-10000 which is due to less amount of PBI incorporated in the backbone, leading to weaker driving force for assembly.

**Figure 3.**
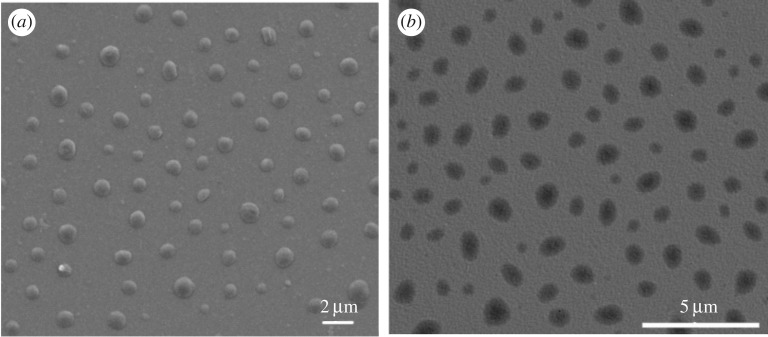
SEM and TEM images of PU-4000 (*a*,*b*) assembled on silicon wafer from 1.0 mg ml^−1^ mixed solutions of toluene and hexane ( 3 : 1, v/v).

To study the concentration effect on morphologies, 1.0 mg ml^−1^ is selected according to the optical spectra results. Figure 3 shows self-assembled morphologies of PU-4000 after treatment with the same mixed solvent of toluene and hexane. SEM (figure 3*a*) shows that PU-4000 presents uniform spherical structure with a diameter of approximately 820 nm. Compared with the concentration at 0.5 mg ml^−1^, the size becomes larger, indicating stronger driving force as the concentration increases. The probable reason could be that the higher concentration of polymer leads to the closer proximity of PBI molecules. Thus, more seeds generate at the initial stage of assembly. After that, the nearby molecules only grow on the formed seeds with a stable solvent evaporation rate, leading to uniform and bigger nanoparticles. TEM shows the formation of core–shell structure in PU-4000 as shown in figure 3*b*. The different contrast is possibly due to different conductivity of the aliphatic chains and PBI segments. As aromatic molecule, PBI is more conductive and hydrophobic than the aliphatic chains. Driven by the π–π stacking and hydrophobicity, the PBI segments exposed to high polarity solvent will stack first, leaving aliphatic chains outside. During the evaporation of hexane, all the aliphatic chains will wrap the PBI segments, forming the core–shell structures.

**Figure 4.**
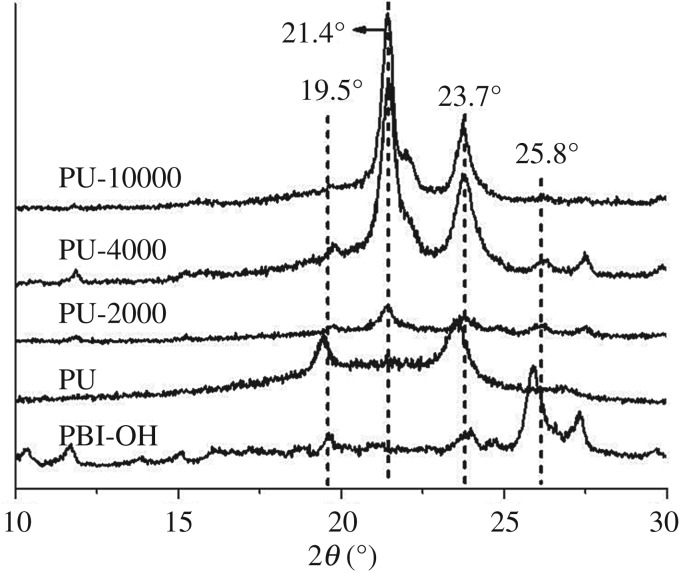
X-ray diffraction patterns of PBI-OH, PU, PU-2000, PU-4000 and PU-10000.

To further verify the role of PBI, XRD is used to collect the diffraction peaks of the assemblies on glass sheets (figure 4). During the preparation of the samples, we dropped the sample solution onto the surface of glass sheets and leaving solvent to evaporate at room temperature. After repeated several cycles, the polymer covered the glass surface with certain thickness. For the neat PU and PBI-OH, XRD curves display two sharp diffraction peaks at approximately 19.3° and 19.5° indicating certain crystallinity. Compared with PU and PBI-OH at 19.3°, different PBI content on the structure could be quantified by the peak areas of the diffraction peaks which are proportional to the linked PBIs in a polymer chain. The diffraction peak at 25.8° shows a well-defined signal in PBI-PUs and PBI-OH with *d*-spacing of 3.4 Å which is a typical π–π stacking distance [27], and π–π stacking between PBI units diminished by incorporating PBI in the PU chains which is shown by the significant reduction in the scattering intensity of the diffraction peak at approximately 25.8°. The intensity decreased as the PBI content decreased from PU-2000, PU-4000 to PU-10000. Furthermore, XRD patterns of PBI-PUs show strong reflections at 21.4° and 23.7°, which are assigned to (110) and (200) refraction of PCL [28]. The intensity was found to increase from PU-2000, PU-4000 to PU-10000 which is due to a higher ratio of PCL.

Meanwhile, the physical properties of solvents are also considered. As shown in electronic supplementary material, table S2, toluene is used as a good solvent with higher polarity and boiling temperature. Hexane is selected as a poor solvent with low polarity and boiling temperature. The assembly mechanism could be that PBI drives aggregation of polymer chains with the assistance of solvents. Initially, the solution of PBI-PUs dropped onto silicon wafer is homogeneous. Hexane occupies certain space on the substrate. As the solvent evaporates, hexane molecules volatilize, leaving homogeneously distributed polymer droplets in toluene. Then, the π–π stacking force of PBIs pulls the nearby polymer chains together, forming spherical cores. During the evaporation of toluene, all the aliphatic chains will wrap the PBI segments, forming the core–shell structures. Nanoparticles are assembled in the process of solvent evaporation.

**Figure 5.**
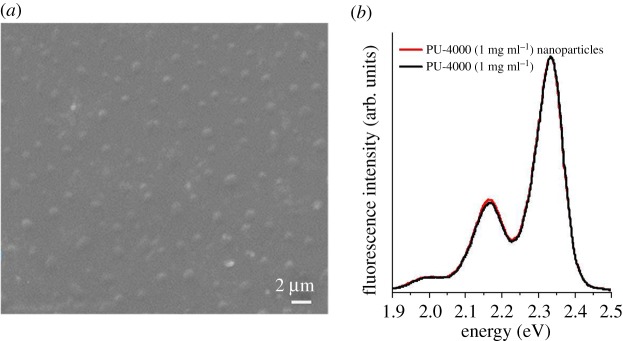
SEM image of PU-4000 assemblies on quartz (*a*). Fluorescence spectra of PU-4000 and PU-4000-nanoparticles on quartz (*b*).

To exclude the substrate effect, glass sheets and quartz are also used to observe the morphologies of the assemblies. PU-4000 with the concentration of 0.5 mg ml^−1^ is selected as an example. SEM (electronic supplementary material, figure S9) shows that the morphologies of assemblies on glass slide are consistent with the morphologies on silicon wafer. A sample on quartz plate is also prepared. The PU-4000 still assembles into uniform nanoparticles with diameter approximately 820 nm on quartz shown in figure 5*a*. The result indicates that the assemblies are not induced from the lattice structure of the silicon wafer. Here, we convert the fluorescence spectral red shift to energy units shown in figure 5*b*, which can be calculated according to the following equation: *E* = *h*/*k* × *C*/*λ*. The value of *h*, *k* and *C* is 6.63 × 10^−34 ^J s, 1.6 × 10^−19^ J eV^−1^ and 3 × 10^17^ nm s^−1^. *λ* represents the wavelength of samples. Figure 5*b* shows that the difference between PU-4000 (2.17 eV) and PU-4000 nanoparticles (2.16 eV) is 0.01 eV, indicating PU-4000 retained optical properties in the assemblies.

On the one hand, UV–vis spectra of PU-4000 film cast from 1.0 mg ml^−1^ THF solution were compared with the corresponding assemblies as shown in electronic supplementary material, figure S10. A red shift of 9 nm was observed in the assemblies. On the other hand, the UV–vis spectra exhibited 12 nm red shift between solution and assembly state as shown in electronic supplementary material, figures S5A and S10 at the concentration of 1.0 mg ml^−1^. The red shifts from solution to film and assemblies indicate more efficient stacking of polymer chains in nanoparticles. Furthermore, the assemblies can be prepared on a large scale of 100 cm and good dispersity as shown in electronic supplementary material, figures S11–S13, which are coincident with the TEM and SEM results.

The results strongly suggest that the morphologies and sizes of nanostructures fabricated from PBI-PUs are dependent on the polymer concentrations, solvent and the ratio of PBI incorporated. It is reported that different forms of nanostructures could be obtained by controlling the evaporation rate of solvents [29–31]. We have tried different solvent systems and selected toluene/hexane = 3 : 1 (v : v) to adjust the evaporation rate. In summary, at the high ratio of PBI, the rigidity of polymer increased. PU-2000 formed crystal-like spindle structure, whereas others formed spherical and soft semi-spherical morphologies at same concentration. Higher concentration of polymer leads to the closer proximity of PBI molecules from which π–π stacking will benefit. The PU-4000 assembled to more uniform particles at higher concentration with stronger driving force as shown in figures 2 and 3.

### 3.4. Thermal stability

**Figure 6.**
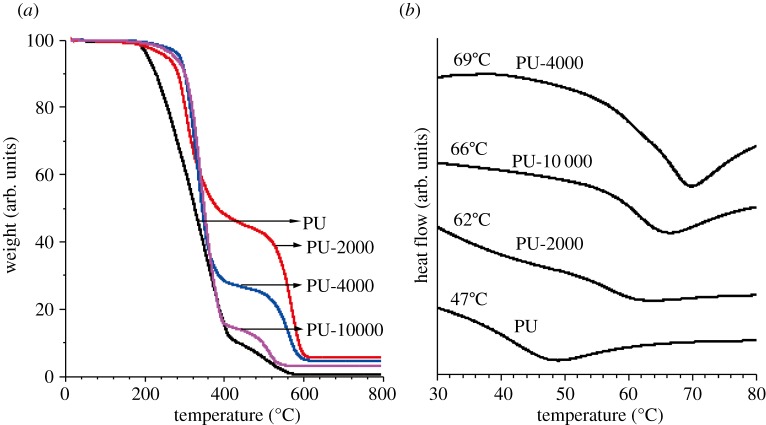
TGA thermograms of PU, PU-2000, PU-4000 and PU-10000 (*a*) and DSC thermograms of PU, PU-2000, PU-4000 and PU-10000, the values indicating the respective glass transition temperatures (*b*).

Thermal stability of the polymers is investigated by TGA and DSC thermograms. The degradation temperatures are shown to be 207°C, 255°C, 286°C and 275°C for neat PU, PU-2000, PU-4000 and PU-10000, respectively, indicating obvious thermal stability of the PBI-PUs compared with neat PU (figure 6*a* and electronic supplementary material, table S1). The temperature corresponding to 5 wt% loss of polymer is regarded as the degradation temperature (*T*_d_). The incorporation of PBIs in the main chains of the PUs obviously retards the random-chain scission and long chain length of modifier enhances the thermal stability further by better dispersion [4,32]. Copolymers and neat PU display similar TGA profiles, showing that the incorporation of PBIs into the main chains of PUs does not obviously change the degradation mechanism. The neat PU and PBI-PUs show two stages of degradation which are oxidation and decomposition of organic substrates in air. In addition, the residues of decomposition of PBI-PUs are higher than the neat PU with increasing content of PBI. The initial decomposition temperatures and the yields of degradation suggest that thermal stabilities of the PBI-PUs are improved. Owing to the lowest oxygen content of PU-2000 (electronic supplementary material, table S3), the amount of residues is the maximum. Elemental analysis results exhibit residues of PBI-PUs which are consistent with the content of oxygen.

DSC thermograms in figure 6*b* show endothermic peaks corresponding to the glass transition temperature at 47°C, 62°C, 69°C and 66°C for PU, PU-2000, PU-4000 and PU-10000 (electronic supplementary material, table S1), respectively. The glass transition temperatures (*T*_g_) varied with polymers with different PBI ratios. Compared with neat PU, PBI-PUs exhibit enhanced *T*_g_. The introduction of PBIs in the main chains of the PUs could increase the rigidity of PUs obviously and thus limit motion of the molecular chains. Consequently, the *T*_g_ of the PBI-PUs is enhanced.

## 4. Conclusion

In summary, amphiphilic PBI-PUs were successfully obtained by the ring-opening reaction and the condensation method. Different sizes of PU nanoparticles (490 nm, 820 nm and 2.1 µm) are obtained via PBI assisted self-assembly. A morphological study shows that the assembly is not only affected by the concentration and structure of the polymer, but also influenced by the solvent evaporation balance which governs the initial space and distributions. Interestingly, the fluorescent properties of PBI are not affected by the particles due to PCL chains. Furthermore, the prepared PUs exhibit enhanced thermal stability because of the existence of PBI. This study provides a new way to fabricate well-defined nanostructures of functionalized PUs and assemblies of polymers which may hold exciting potential in the field of drug delivery and biological labelling.

## Supplementary Material

Supporting figures Figure S1 to Figure S12

## Supplementary Material

Supporting table Table S1 to Table S3

## Data accessibility

The datasets supporting this article have been uploaded as part of the electronic supplementary material.
