# Supplementary material for: Nanostructured polyurethane perylene bisimide ester assemblies with tuneable morphology and enhanced stability
Source: R Soc Open Sci. 2018 Mar 21;5(3):171686. doi: 10.1098/rsos.171686 (PMC5882699; doi:10.1098/rsos.171686)
Supplement: Supporting figures Figure S1 to Figure S12 [file rsos171686supp1.docx]

Nanostructured polyurethane perylene bisimide ester assemblies with tunable morphology and enhanced stability

Xiaoxiao Zhang, Tingyuan Gong, Hong Chi*, Tianduo Li*

Shandong Provincial Key Laboratory of Fine Chemicals, School of Chemistry and Pharmaceutical Engineering, Qilu University of Technology, Jinan 250353, People's Republic of China

Corresponding Author: Hong Chi and Tianduo Li, Shandong Provincial Key Laboratory of Fine Chemicals, School of Chemistry and Pharmaceutical Engineering, Qilu University of Technology, Jinan 250353, People's Republic of China, [ch9161@gmail.com](mailto:ch9161@gmail.com); [litianduo@163.com](mailto:litianduo@163.com).

Supplementary Material: Figures S1-13


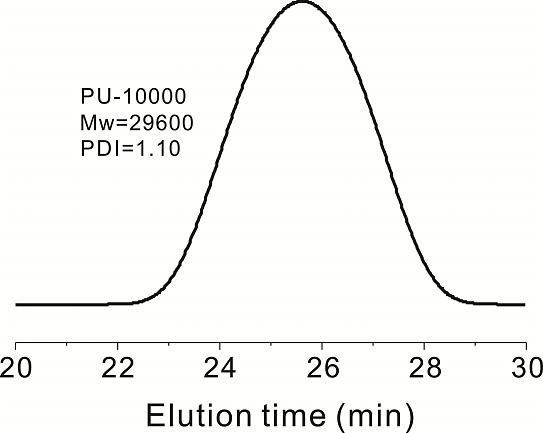


**Figure S1.** GPC trace of PU-10000. The GPC trace of PU-10000 is selected as an example which shows narrow molecular weight distribution.


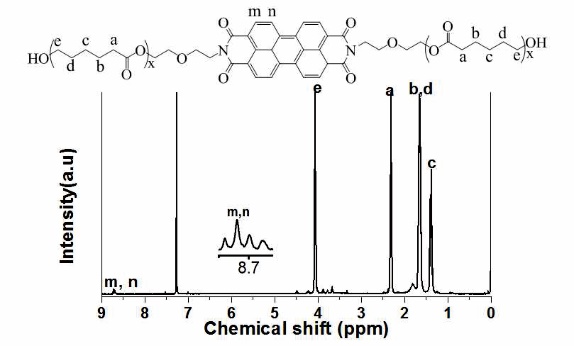


**Figure S2.** ^1^H NMR spectrum of O-2000 in CDCl_3_. The peaks of ^1^H NMR spectrum of O-2000 appeared at 4.1 ppm (e), 2.3 ppm (a), 1.6 ppm (b, d), 1.4 ppm (c) are assigned to CH_2_ on poly (ε-caprolactone) (PCL), suggesting PCL is grafted onto PBI-OH successfully.


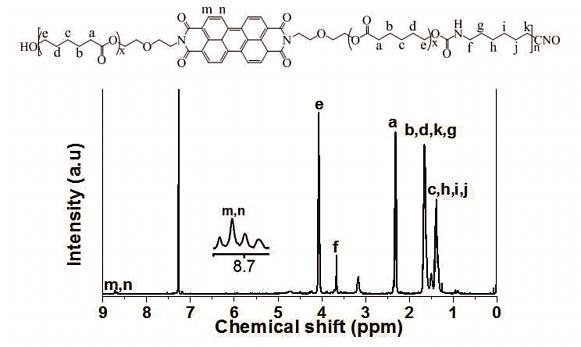


**Figure S3.** ^1^H NMR spectrum of PU-2000 in CDCl_3_. The signals of PU-2000 appeared at 1.4 ppm (h, i, j), 1.6 ppm (k, g) and 3.6 ppm (f) are assigned to CH_2_ protons in HDI. The typical aromatic protons of PBI appear at 8.6-8.8 ppm in the polymers, indicating that the PBI moieties have been successfully incorporated into the main chain.


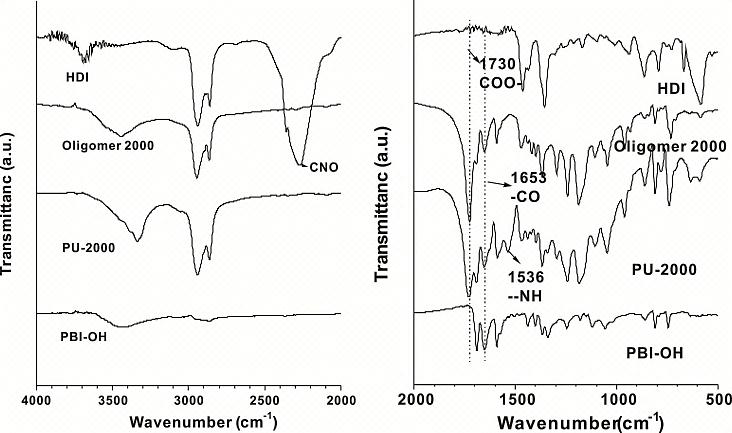


**Figure S4.** FTIR spectra of PBI-OH, HDI, PU-2000, O-2000. FTIR of PU-2000 showing the peak of -NCO in HDI at 2260 cm^-1^ disappeared after polymerization. The strong band at 1653 cm^-1^ is attributed to the CO vibrations in PBI-OH and is recognizable in oligomer and polymer. The appearance of vibrational peaks of -COO at 1730 cm^-1^ and the -NH vibrations at 1536 cm^-1^ further confirm the successful polymerization.


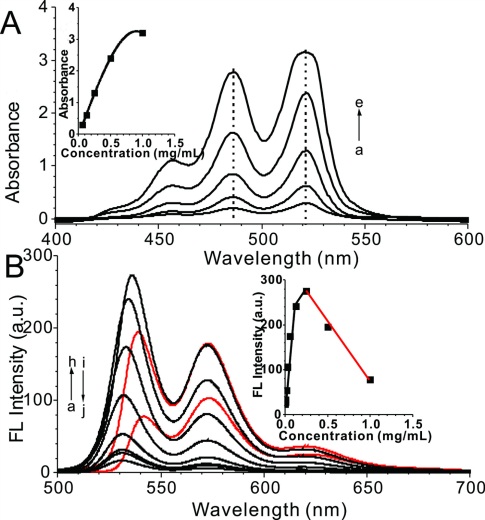


**Figure S5.** UV-vis absorption spectra (A) of THF solutions of PU-4000 at concentrations (a to e) of 0.06 mg/mL, 0.125 mg/mL, 0.25 mg/mL, 0.5 mg/mL and 1.0 mg/mL. The linear increasing tendency suggests that the solubility of PBI is greatly improved and PU-4000 can restrain the aggregation of PBI cores through side chain effect. Fluorescence spectra (B) of THF solutions of PU-4000 at concentrations (a to j) of 0.0015 mg/mL, 0.003 mg/mL, 0.006 mg/mL, 0.015 mg/mL, 0.03 mg/mL, 0.06 mg/mL, 0.125 mg/mL, 0.25 mg/mL, 0.5 mg/mL, 1.0 mg/mL. The fluorescence intensity increases linearly with the concentration ranging from 0.0015 mg/mL to 0.25 mg/mL. The intensity persistently decreases when further increase concentration from 0.5 mg/mL to 1.0 mg/ mL. The reason is that self-quenching and π-π stacking interactions occurring in the perylene cores, and the peaks are gradually shifted to the red with increasing concentration because of re-absorption. Interestingly, the peak only red-shift 7 nm, indicating that the optical property is retained in PU-4000.


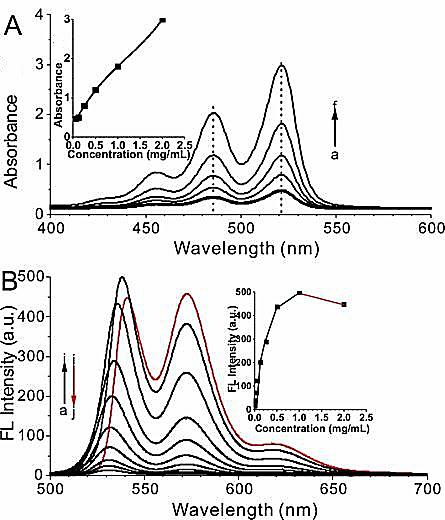


**Figure S6.** UV-vis absorption spectra (A) of THF solutions of PU-10000 at concentrations (a to f) of 0.06 mg/mL, 0.125 mg/mL, 0.25 mg/mL, 0.5 mg/mL, 1.0 mg/mL and 2.0 mg/mL. The linear increasing tendency suggests that the solubility of PBI is greatly improved and PU-10000 can restrain the aggregation of PBI cores through side chain effect. Fluorescence spectra (B) of THF solutions of PU-10000 at concentrations (a to j) of 0.003 mg/mL, 0.006 mg/mL, 0.015 mg/mL, 0.03 mg/mL, 0.06 mg/mL, 0.125 mg/mL, 0.25 mg/mL, 0.5 mg/mL, 1.0 mg/mL, 2.0 mg/mL. The fluorescence intensity increases linearly with the concentration ranging from 0.003 mg/mL to 1.0 mg/mL. The intensity persistently decreases when further increase concentration from 1.0 mg/mL to 2.0 mg/ mL. The reason is that self-quenching and π-π stacking interactions occurring in the perylene cores, and the peaks are gradually shifted to the red with increasing concentration because of re-absorption. Interestingly, the peak only red-shift 5 nm, indicating that the optical property is retained in PU-10000.


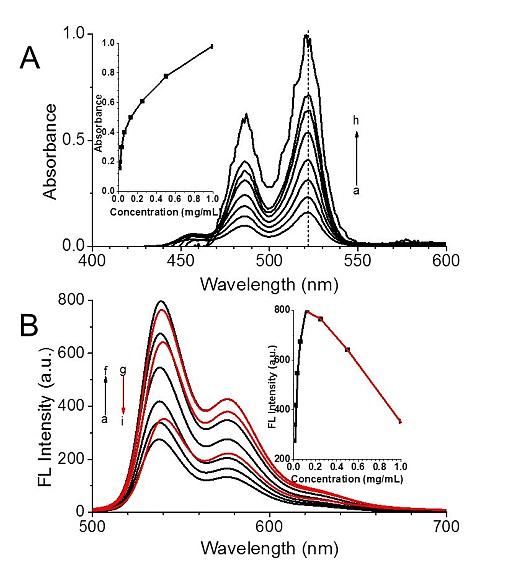


**Figure S7.** UV-vis absorption spectra (A) of THF solutions of PBI-OH at concentrations (a to h) of 0.006 mg/mL, 0.015 mg/mL, 0.03 mg/mL, 0.06 mg/mL, 0.125 mg/mL, 0.25 mg/mL, 0.5 mg/mL and 1.0 mg/mL. The linear increasing tendency suggests that the solubility of PBI is greatly improved. Fluorescence spectra (B) of THF solutions of PBI-OH at concentrations (a to j) of 0.003 mg/mL, 0.006 mg/mL, 0.015 mg/mL, 0.03 mg/mL, 0.06 mg/mL, 0.125 mg/mL, 0.25 mg/mL, 0.5 mg/mL and 1.0 mg/mL. The fluorescence intensity increases linearly with the concentration ranging from 0.003 mg/mL to 0.125 mg/mL. The intensity persistently decreases when further increase concentration from 0.125 mg/mL to 1.0 mg/ mL. The reason is that self-quenching and π-π stacking interactions occurring in the perylene cores, and the peaks are gradually shifted to the red with increasing concentration because of re-absorption. UV-vis shows no obvious bathochromic shift and FL shows that the peak only red-shift 5 nm with the increase of concentration. The UV-vis and FL of PBI-OH are similar to these of the PU-2000, PU-4000 and PU-10000 which further prove that there are no aggregation of PBI-OH and polymers in THF solution.


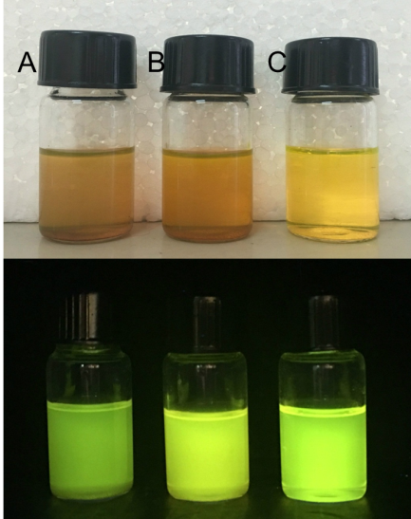


**Figure S8.** Photographs of PU-2000 (A), PU-4000 (B) and PU-10000 (C) in THF solution with the concentration of 1.0 mg/mL without and with 365 nm UV excitation. All solutions exhibit bright greenish yellow fluorescence color under UV excitation. PU-10000 is the brightest because the long and branched PCL chains could effective separate PBI.


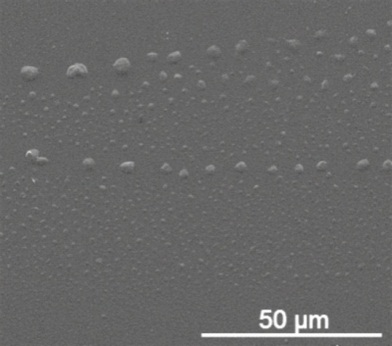


**Figure S9.** SEM image of PU-4000 (0.5 mg/mL) assembled on glass substrate from mixed solutions of toluene and hexane. It shows that polymers spontaneously form uniform spherical structures and the diameters are about 490 nm and 2.1 μm in alternated arrangement on glass slide which are consistent with the morphologies on silicon wafer.


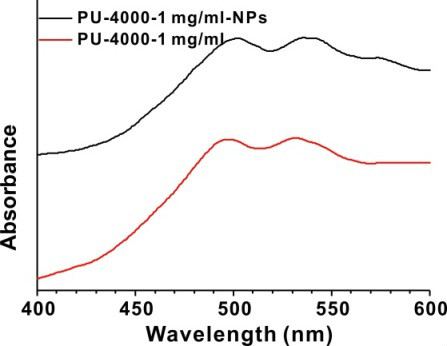


**Figure S10.** UV-vis absorption spectra of PU-4000-1.0 mg/mL and PU-4000-1.0 mg/mL-NPs on quartz. UV-vis spectra show only 9 nm red-shift. The probable reason is that long molecular chain of polymer could effectively separate PBI both intermolecularly and intramolecularly.


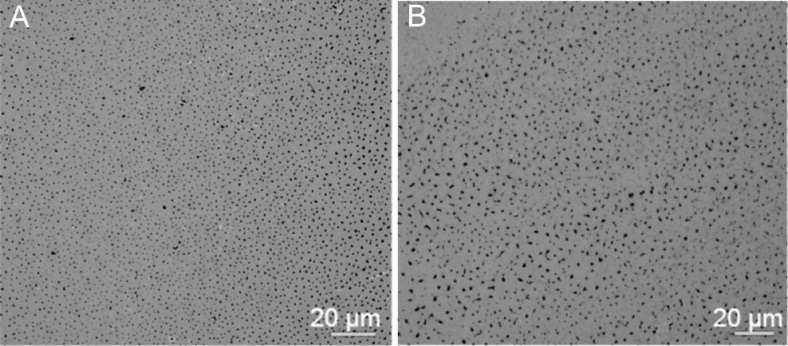


**Figure S11.** Optical microscopic images of PU-2000 assembled on silicon wafer from mixed solutions of toluene and hexane, (A) 0.5 mg/mL, (B) 1.0 mg/mL. The assemblies of PU-2000 can be prepared in large scale and good dispersity. Compared with the concentration at 0.5 mg/mL, the size becomes larger, indicating stronger driving force as the concentration increase.


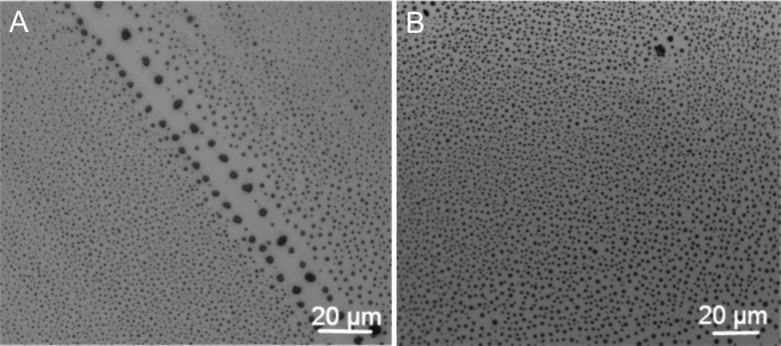


**Figure S12**. Optical microscopic images of PU-4000 assembled on silicon wafer from mixed solutions of toluene and hexane, (A) 0.5 mg/mL, (B) 1.0 mg/mL. The assemblies of PU-4000 can be prepared in large scale and good dispersity. Compared with the concentration at 0.5 mg/mL, the size becomes larger, indicating stronger driving force as the concentration increase.


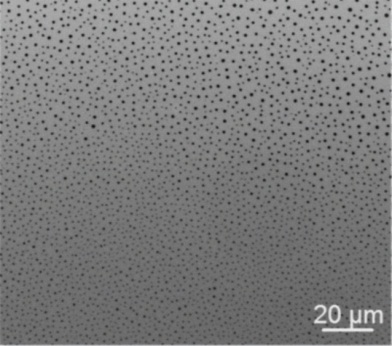


**Figure S13.** Optical microscopic images of PU-10000 assembled on silicon wafer from mixed solutions of toluene and hexane. The assemblies of PU-10000 can be prepared in large scale and good dispersity.
