# Supplementary material for: Nanostructured polyurethane perylene bisimide ester assemblies with tuneable morphology and enhanced stability
Source: R Soc Open Sci. 2018 Mar 21;5(3):171686. doi: 10.1098/rsos.171686 (PMC5882699; doi:10.1098/rsos.171686)
Supplement: Supporting table Table S1 to Table S3 [file rsos171686supp2.docx]

Nanostructured polyurethane perylene bisimide ester assemblies with tunable morphology and enhanced stability

Xiaoxiao Zhang, Tingyuan Gong, Hong Chi*, Tianduo Li*

Shandong Provincial Key Laboratory of Fine Chemicals, School of Chemistry and Pharmaceutical Engineering, Qilu University of Technology, Jinan 250353, People's Republic of China

Corresponding Author: Hong Chi and Tianduo Li, Shandong Provincial Key Laboratory of Fine Chemicals, School of Chemistry and Pharmaceutical Engineering, Qilu University of Technology, Jinan 250353, People's Republic of China, [ch9161@gmail.com](mailto:ch9161@gmail.com); [litianduo@163.com](mailto:litianduo@163.com).

Supplementary Material: Tables S1-3

**Table S1.** Characteristics of PU, PU-2000, PU-4000 and PU-10000. GPC shows narrow molecular weight distribution in all the PBI-PUs ranging from 1.03 to 1.10. This is possibly due to the ring-opening reaction from ε-CL which possesses unique monomer length and reactive sites. Additionally, PU-4000 displays the highest molecular weight which is probably due to the high steric hindrance when higher ratio of PBI or longer ε-CL chains is used. T_d_ and T_g_ are also displayed in the table.

| Polyurethane | M_w_ | M_n_ | M_w_/M_n_ | T_d_ (℃) | T_g_ (℃) |
| --- | --- | --- | --- | --- | --- |
| PU | 22600 | 22100 | 1.02 | 207 | 47 |
| PU-2000 | 22800 | 22200 | 1.03 | 255 | 62 |
| PU-4000 | 31500 | 28700 | 1.09 | 286 | 69 |
| PU-10000 | 29600 | 26700 | 1.10 | 275 | 66 |

**Table S2**. The solvent parameters of toluene and hexane. Assembled morphologies are influenced by the solvent evaporation balance which governs the initial space and distributions. Here toluene is used as a good solvent with higher polarity and boiling temperature. Hexane is selected as poor solvent with low polarity and boiling temperature.

| Solvent | Polarity index | Boiling point (℃) | Evaporation rate (ethyl acetate=100) |
| --- | --- | --- | --- |
| Toluene | 2.4 | 110 | 230 |
| Hexane | 0 | 69 | 280 |

**Table S3.** Elemental analysis of PU, PU-2000, PU-4000, PU-10000. Due to the lowest oxygen content of PU-2000, the amount of residues is the maximum. Elemental analysis results exhibit residues of PBI-PUs which are consistent with the content of oxygen.

| Polyurethane | C | N | O | H |
| --- | --- | --- | --- | --- |
| PU | 54.0% | 2.5% | 38.7% | 4.8% |
| PU-2000 | 58.0% | 4.0% | 26.1% | 11.9% |
| PU-4000 | 57.8% | 2.3% | 27.0% | 12.9% |
| PU-10000 | 59.6% | 0.8% | 28.4% | 11.2% |
